# Autocrine IL-6/STAT3 signaling aids development of acquired drug resistance in Group 3 medulloblastoma

**DOI:** 10.1038/s41419-020-03241-y

**Published:** 2020-12-05

**Authors:** Lakshana Sreenivasan, Hui Wang, Shyong Quin Yap, Pascal Leclair, Anthony Tam, Chinten James Lim

**Affiliations:** 1 Department of Medicine, University of British Columbia, Vancouver, BC Canada; 2 Michael Cuccione Childhood Cancer Research Program, BC Children’s Hospital Research Institute, Vancouver, BC Canada; 3 Department of Pediatrics, University of British Columbia, Vancouver, BC Canada; 4 Department of Microbiology and Immunology, Jinan University, Guangzhou, People’s Republic of China

**Keywords:** Cancer microenvironment, CNS cancer, Paediatric cancer

## Abstract

Medulloblastoma (MB) is a high-grade pediatric brain malignancy that originates from neuronal precursors located in the posterior cranial fossa. In this study, we evaluated the role of STAT3 and IL-6 in a tumor microenvironment mediated drug resistance in human MBs. We established that the Group 3 MB cell line, Med8A, is chemosensitive (hence Med8A-S), and this is correlated with a basal low phosphorylated state of STAT3, while treatment with IL-6 induced robust increases in pY705-STAT3. Via incremental selection with vincristine, we derived the stably chemoresistant variant, Med8A-R, that exhibited multi-drug resistance, enhanced IL-6 induced pY705-STAT3 levels, and increased IL6R expression. Consequently, abrogation of STAT3 or IL6R expression in Med8A-R led to restored chemosensitivity to vincristine, highlighting a prominent role for canonical IL-6/STAT3 signaling in acquired drug resistance. Furthermore, Med8A-S subjected to conditioning exposure with IL-6, termed Med8A-IL6+ cells, exhibited enhanced vincristine resistance, increased expression of pY705-STAT3 and IL6R, and increased secretion of IL-6. When cocultured with Med8A-IL6+ cells, Med8A-S cells exhibited increased pY705-STAT3 and increased IL-6 secretion, suggesting a cytokine feedback loop responsible for amplifying STAT3 activity. Similar IL-6 induced phenomena were also observed in the Group 3 MB cell lines, D283 and D341, including increased pY705-STAT3, drug resistance, IL-6 secretion and IL6R expression. Our study unveiled autocrine IL-6 as a promoter of STAT3 signaling in development of drug resistance, and suggests therapeutic benefits for targeting the IL-6/STAT3 signaling axis in Group 3 MBs.

## Introduction

Medulloblastoma (MB) is a high-grade pediatric brain malignancy that originates from neuronal precursor cells located in the posterior cranial fossa. It is the most common type of brain tumor diagnosed in children with an overall 5-year survival rate of 60–80%, depending on the risk classification. MB was once known as a single disease entity that has since been classified into four distinct molecular variants based on clinical heterogeneity and transcriptome array profiling. The WHO classification of the four molecular subgroups of MB- Wingless (WNT), sonic hedgehog (SHH), group 3, and group 4, demands specific molecular targeted therapies to treat each subgroup distinctly to improve the prognosis of the disease^1,2^.

MB is primarily treated with a combination of conventional therapies including surgery, craniospinal irradiation, and cytotoxic chemotherapy (commonly vincristine, cisplatin, and cyclophosphamide)^3^. While intensification of non-specific conventional therapies has led to significant improvements in patient survival, this achievement is accompanied with more severe long-term sequelae of survivors. As MB more commonly afflicts infants and children under 4 years old, aggressive chemotherapies also lead to harmful side-effects including cognitive impairment, endocrine disorders, and increased incidence of secondary cancers later in life^4,5^.

The current clinical implications of molecular subgroups need to be considered to improve the quality of treatment for MB patients. Among the four molecular subgroups, Group 3 has been histologically and molecularly classified as the most aggressive type of MB, with metastases found in most patients at the time of diagnosis. Group 3 MB has the worst overall survival (below 50%), is more common in males, and predominantly found in infants and children^6^. Transcriptomic studies have revealed high levels of MYC amplification in this subgroup. Group 3 MB is known to originate from cerebral stem cells located in the external granular layer and rhombic lip region of the cerebellum. Due to the blood-brain barrier and the location of the tumor, MB is one of the most challenging tumors to treat^7^. Chemotherapy and radiation, still the mainstay of MB treatments, is limited by therapeutic resistance^1,8–10^.

Signal transducer and activator of transcription (STAT) proteins are often implicated in tumorigenesis of multiple malignancies^11–13^. Of the seven STATs, STAT3, and STAT5 have been known to play a predominant role in tumor cell proliferation and survival. Increased STAT3 phosphorylation, which occurs downstream of Janus activated kinases (JAK), is commonly associated with drug-resistant recurrent tumors, when compared to primary tumors^14^. STAT3 is also required for the recruitment of stromal and immune cells to the tumor microenvironment to facilitate tumor progression^15^.

Interleukin 6 (IL-6) is a potent pleiotropic cytokine that plays a functional role in tumorigenesis. Indeed, high levels of serum IL-6 was correlated with shortened duration of overall survival in multiple malignancies^16^. IL-6 is also the best-established upstream regulator and activator of STAT3; IL-6 binding to its receptor IL-6Rα induces dimerization of a receptor complex that includes gp130 (a common β subunit for IL-6 family of cytokine receptors). This leads to proximity mediated transactivation of the gp130-associated JAKs, and subsequent phosphorylation and dimerization of cytoplasmic STAT3, which then translocates into the nucleus to complex with other nuclear proteins that bind DNA and control target gene expression. Whereas STAT3 is transiently phosphorylated in response to IL-6 stimulation in normal cells, constitutive activation of STAT3 are often implicated in treatment-refractory cancers^17^. As nuclear transcription factors that act to switch on expression of pro-survival and oncogenic proteins downstream of cytokine (growth factor) signaling, the IL-6/STAT3 pathway represents a promising molecular target for therapy.

IL-6 stimulation of JAK/STAT3 signaling can occur via an autocrine (cell response to its own secreted signal) or paracrine (signal secreted by other cells in the tumor microenvironment) manner, subsequently contributing to cellular growth and transformation^18^. In this study, we focus on the preclinical approaches that help predict the emergence of drug resistance via IL-6/STAT3 autocrine signaling in human Group 3 MB. Using incremental drug selection, we generated chemoresistant MB cell lines with enhanced IL-6/STAT3 activity. Conversely, cytokine conditioning without drug pre-exposure was sufficient to convert chemosensitive MB cells to a chemoresistant variety. The critical requirement of the IL-6/STAT3 signaling axis in MB cell survival and drug resistance was evaluated using CRISPR-Cas9 engineered cells with loss of IL-6R or STAT3 function. Our study highlights the pro-tumorigenic role of IL-6/STAT3 signaling, and implicate the potential for molecular targeted therapies to counter acquired drug resistance in Group 3 MB.

## Materials and methods

### Cells and tissue culture

MED-MEB-8A^19^, referred to as Med8A-S, and derived variants were cultured in 10% fetal bovine serum (FBS, Invitrogen) DMEM (Sigma) supplemented with 1% penicillin–streptomycin (Pen-Strep, Gibco) and non-essential amino acids (NEAA, Invitrogen). Med8A-R cells was derived from Med8A-S by incremental selection over a period of 4 months with vincristine (Sigma) at concentrations starting at 0.01 µg/mL and ending at 0.16 µg/mL. Med8A-S and Med8A-R were confirmed with identical STR profiles (ATCC).

D341 Med (ATCC HTB­187™) and D283 Med (ATCC HTB­185™) were cultured in 20% FBS EMEM (Sigma) and 10% FBS EMEM, respectively, while DAOY (ATCC HTB-186) was cultured in 10% FBS DMEM, all with 1% Pen-Strep and NEAA. D341, D283, and DAOY were purchased from ATCC, and used for experiments within passages 3–12.

IL-6 conditioning of cells (denoted as IL6+) was achieved by culturing each cell line for 4 weeks in media supplemented with 2 ng/mL recombinant human IL-6 (Genscript). Following this conditioning, cells were cultured a further 2 weeks without IL-6 addition before being used for experiments.

For conditioning using the coculture system, we refer to target cells as ones being conditioned, while donor cells represent the source of stimulatory cytokines. Target cells are plated at a density of 10^5^ cells/well in a 6-well plate. Donor cells (e.g., IL6+ cells already pre-conditioned with IL-6), are plated at a density of 10^5^ cells within a transwell insert (Greiner ThinCert) that is then placed into the well to coincubate with the target cells in fresh culture media (with no added cytokine). Following the coculture for 3 days, the transwell insert is removed, the target cells rinsed with blank media, and replenished with fresh media for another 3 days. Cells were either harvested for protein analysis, or the media supernatant analyzed for secreted cytokines.

### Cell viability assays

Cells were seeded in 96-well plates at a density of 10^5^ cells/well, allowed to adhere for 16 h before addition of drug/s at various concentrations. After 48 h, the fluorometric reagent Cell Titer Blue (Promega) was added according to manufacturer’s protocol and fluorescence (560_Ex_/590_Em_) measured on a spectrophotometer (Enspire) after 4 h. In addition to vincristine (Sigma), cells were also treated with cisplatin, idarubicin, mitoxantrone, or niclosamide (Selleckchem). All assays were conducted as 3 replicates per treatment condition and graphs were plotted using GraphPad Prism.

### Western immunoblots

Cell lysates were prepared in PN lysis buffer (10 mM PIPES, 50 mM NaCl, 150 mM sucrose, 50 mM NaF, 40 mM Na_4_P_2_O_7_10H_2_O, 1 mM Na_3_VO_4_, 1% Triton X-100, Complete protease inhibitors (Sigma)). Total protein (30 µg) was separated by SDS-PAGE, and transferred to nitrocellulose using the Trans-Blot Turbo Transfer System (Bio-Rad). Blots were blocked in blocking buffer 5% bovine serum albumin (BSA, Fisher) in TBS-T (TBS-T is 50 mM Tris-Cl, 150 mM NaCl, pH 8 with 0.1% Tween20) for 1 hour at 22 ^o^C, then incubated overnight at 4 ^o^C with primary antibodies diluted in blocking buffer. Blots were further incubated with secondary goat anti-mouse or -rabbit fluorophore-conjugated antibodies (Dylight 800 or Dylight 680, ThermoFisher) in 2% non-fat milk in TBS-T, and scanned on the Licor Odyssey.

The following primary antibodies were used: pY705-STAT3, pS727-STAT3, and STAT3 (Cell Signaling Technologies); and GAPDH (Biolegend). In some experiments, cells were treated with IL-6 at the indicated concentrations and time prior to preparation of cell lysates.

### Plasmids and CRISPR

Guide RNA (gRNA) mediated CRISPR-Cas9 gene editing was used to generate null cell lines. To target exon 2 of *STAT3*, DNA corresponding to 5′ GCAGGAAGCGGCTATACTGC 3′ gRNA sequence was cloned into plasmid pX330 (Addgene #42230). To target exon 1 of *IL6Rα*, 5′ GGCCGTCGGCTGCGCGCTGC 3′ was cloned into pX458 (Addgene #48138). Med8A-R cells were transfected with the respective plasmids using Lipofectamine 2000 (ThermoFisher) as per manufacturer’s instructions. After 1 day, cells were clonally sorted by flow into 96-well plates (FacsAria, BD Biosciences). Screening for STAT3^−/−^ clones involved immunofluorescence evaluation for STAT3 expression, followed by western blot confirmation. Screening for IL6Rα^−/−^ clones was done by flow cytometry. To identify indel mutations within the targeted genomic loci, we sequenced a genomic amplicon generated by polymerase chain reaction using the following primers: 5′ CACCGGGCCGTCGGCTGCGCGCTGC 3′ (Fwd) and 3′ AAACGCAGCGCGCAGCCGACGGCCC 5′ (Rev) for *STAT3*, and 5′ TTCCTATCAGTGGACCGCGT 3′ (Fwd) and 3′ ACATTGATGGCATTTTATTGCTGA 5′ (Rev) for *IL6Rα*. Sequence alignments of the CRISPR generated mutants and parental strain were performed using CLC Main Workbench to confirm the knockout.

### Flow cytometry

To evaluate cell surface IL6R expression, cells were suspended, washed with phosphate buffered saline and stained with anti-human IL6Rα antibody (R&D Systems) followed by secondary antibody (Dylight 488; ThermoFisher). Flow cytometry was conducted on the Accuri C6 (BD Biosciences) and analysis was conducted using FlowJo (Tree Star). Fluorescence activated cell sorting of CRISPR generated cells was conducted on the FacsAria (BD Biosciences) in the BCCHRI Flow Core facility.

### Real Time PCR

Total RNA was extracted from Med8A-S, Med8A-R, and Med8A-IL6+ cells using the RNeasy mini plus kit (Cat# 74136; Qiagen). 1 µg RNA was reverse-transcribed into cDNA using the iScript cDNA synthesis kit (1708891; Biorad) and real-time PCR was performed using TaqMan Fast Advanced Mastermix (# 4444556, Applied Biosystems) on the ABI StepOne System (Applied Biosystems). Pre-designed human PrimeTime qPCR Probe Assays (Integrated DNA Technologies) was used to measure gene expression of IL6R (Hs.PT.58.3039085) and E2F3 (Hs.PT.58.22827120), and normalized to HPRT1 (Hs.PT.58 v.45621572). The relative changes in target gene were analyzed using the 2^−ΔΔCt^ method.

### Cell proliferation and colony forming assay

Cell proliferation was assessed by seeding 5000 cells per well in a 96-well plate and cell density monitored for 5 days. Colony forming assay was performed by seeding 200 cells per well in a 96-well plate and monitored for 7 days. Both assays were performed and analyzed on the IncuCyte live-cell imaging platform (Sartorius).

### ELISA and cytokine array

IL-6 cytokine secreted by cells was quantified using LEGEND MAX Human IL-6 ELISA Kit (Biolegend #430507). Media supernatant collected from culture of various cells were incubated and stained with reagents on a pre-coated 96-well strip plate as per manufacturer’s instructions. A microplate reader (Enspire) was used to measure the absorbance at 450 nm.

Quantibody^®^ Human Cytokine Array 1 (RayBiotech) is a multiplex ELISA system for quantitative measurement of multiple cytokines simultaneously. Sample preparation and analysis was performed according to the manufacturer’s instructions. Further statistical analysis was performed using GraphPad.

### Data mining of gene expression profiles

Functional genomics data repository available on Gene Expression Omnibus was used to query gene expression profiles of MB patients. Raw Affymetrix CEL files consisting of exon array data of 763 MB primary MB specimens (GSE85217) were extracted to perform gene-level analysis. Gene expression values were downloaded from the SOFT file and gene nomenclature was acquired from the Matrix file. Relevant clinical information regarding age, sex, histology, subgroup classification of MB patients was used in this analysis. STAT3, IL-6, and IL6R gene expression values were consolidated based on their subgroup classifications and plotted using GraphPad.

### Statistical data analysis

All data are representative of at least three independent experiments. Graphs were plotted and statistical significance calculated using GraphPad, with ***p < 0.001, **p < 0.01, *p < 0.05, ns – not significant. The statistical tests used is indicated in each figure legend.

## Results

### Drug resistant MB is correlated with increased IL-6/STAT3 activity

**Fig. 1:**
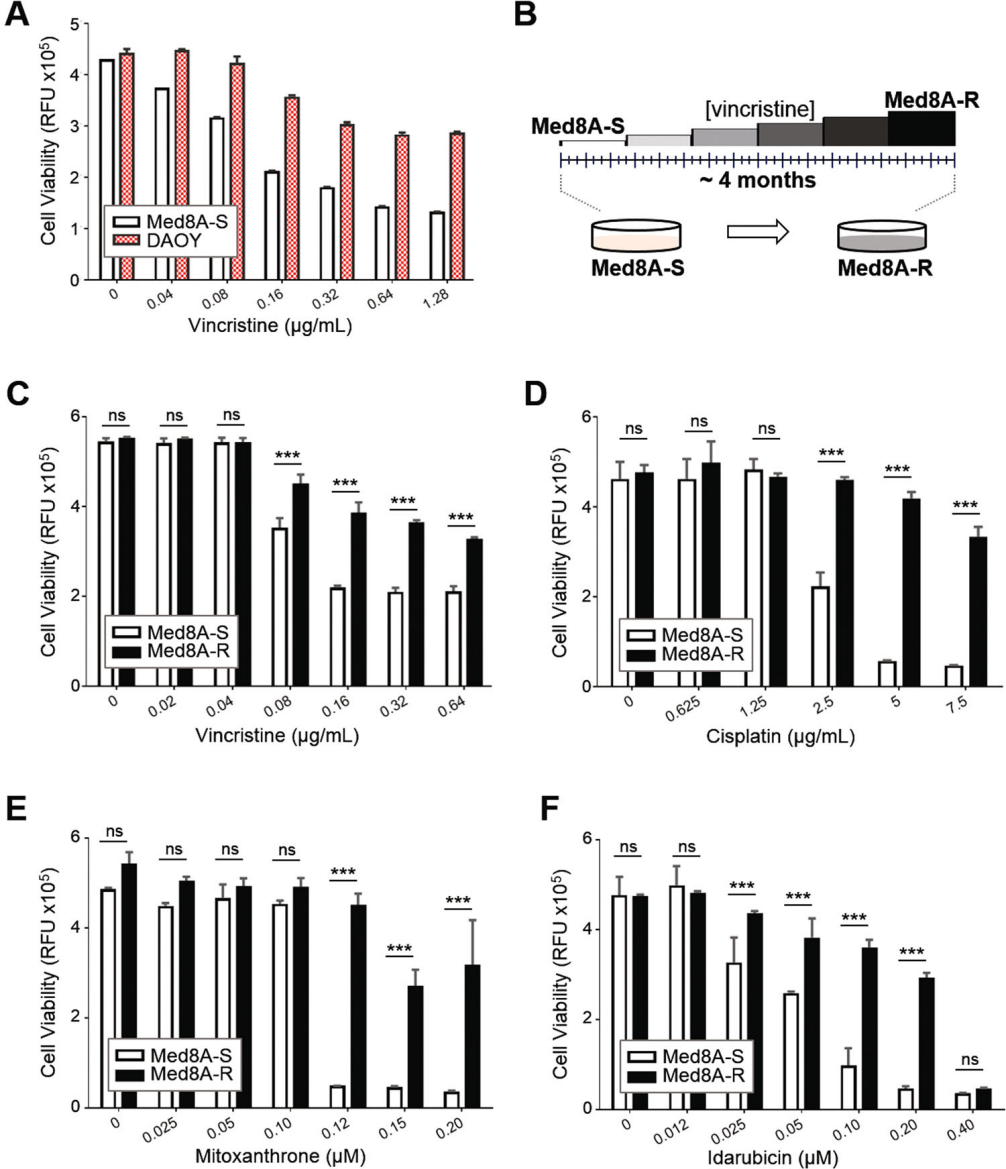
Derivation of a chemoresistant variant of Med8A MB. **a** Med8A-S and DAOY cells were treated with vincristine at the indicated concentrations for 48 h, and cell viability assessed by measuring the fluorescence after incubation with CellTiter-Blue (CTB) for 4 h. As plotted is the mean ± SD of an experiment performed in triplicates, representative of at least 3 independent experiments. **b** Schematic for derivation of drug resistant Med8A cells. The chemosensitive parental cell line, Med8A-S, was subjected to incremental selection in vincristine over a period of 4 months to derive the stable chemoresistant variant Med8A-R. The endpoint vincristine concentration was at 0.16 μg/mL. Med8A-S and Med8A-R were treated with (**c**) vincristine (**d**) cisplatin (**e**) mitoxantrone and (**f**) idarubicin for 48 h and cell viability assessed with CTB. The data represent the mean ± SD of three replicates; ***p < 0.001, two-way ANOVA with Bonferroni’s multiple comparison test.

Initially, we evaluated the sensitivity of two human MB cell lines to vincristine, a vinca alkaloid that destabilizes microtubules and is one of the primary agents for clinical treatment of MB^3,20^. Med8A, which belongs to Group 3 MB^21^, appears to be highly sensitive to vincristine when compared to DAOY, an SHH subgroup MB (Fig. 1a). This presented an opportunity to contrast pathway changes resulting from acquired drug resistance, thus we subjected the chemosensitive parental line (herein referred to as Med8A-S) to gradual incremental selection with vincristine, and derived a stably chemoresistant variant termed Med8A-R (Fig. 1b). Despite being selected with only vincristine, we found that Med8A-R exhibited significant resistance to other agents including cisplatin, mitoxantrone, and idarubicin (Fig. 1c–f).

**Fig. 2:**
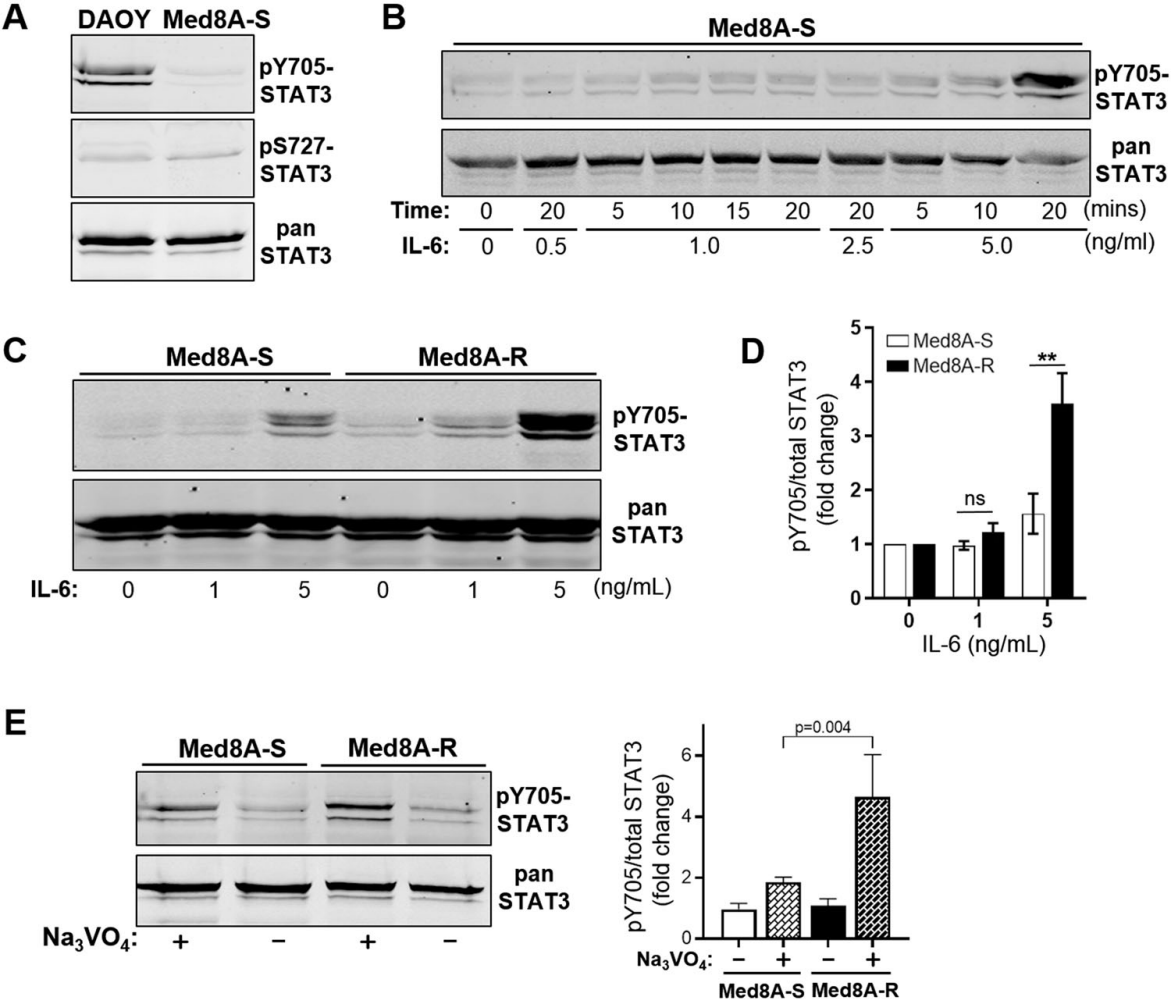
Chemoresistant MB is associated with enhanced STAT3 activity. **a** Lysates of DAOY and Med8A-S cells under basal, nonstimulated conditions were assessed for levels of phosphorylated (pY705 and pS727) and total STAT3 by Western blot analysis. **b** Med8A-S was treated with IL-6 at various concentrations for various times to identify conditions promoting optimal but non-saturating stimulation of pY705-STAT3. **c** Med8A-S and Med8A-R cells were treated with IL-6 at 1 or 5 ng/mL for 10 min and cell lysates immunoblotted for pY705 and total STAT3. As shown is representative of 3 independent replicates. **d** Quantitation of pY705-STAT3 over total STAT3, reflected as fold change, from the data shown in (**c**). **p < 0.01, two-way ANOVA with Bonferroni’s post-test. **e** Med8A-S and Med8A-R cells were treated with 2 mM sodium vanadate for 20 min, and cell lysates immunoblotted for pY705 and total STAT3. Left panel: As shown is representative of 3 independent replicates. Right panel: Quantitation of pY705-STAT3 over total STAT3, reflected as fold change. Significance determined by two-way ANOVA with Bonferroni’s multiple comparison test.

Increased STAT3 activity is known to regulate most tumorigenic functions and promote drug resistance in gliomas^22–24^ and multiple other malignancies^25–27^. To infer the role of STAT3 in drug resistance of MB, we profiled the phosphorylation status at Tyr705 (pY705-STAT3) and Ser727 (pS727-STAT3) in lysates of Med8A and DAOY cells by western blot analysis. Under nonstimulated basal culture conditions, DAOY exhibited higher pY705-STAT3 levels when compared to Med8A-S (Fig. 2a), suggesting that enhanced STAT3 activity may be involved in enhanced drug resistance of DAOY. Next, we titrated the concentration and duration of IL-6 treatment to optimize conditions able to stimulate pY705-STAT3 in Med8A cells in a non-saturating manner (Fig. 2b). Even though nonstimulated Med8A-S and Med8A-R cells expressed comparable levels of STAT3 (Supplementary Fig. 1) and pY705-STAT3, IL-6 treatment invoked a stronger pY705-STAT3 response in Med8A-R compared to Med8A-S (Fig. 2c, d), implicating enhanced sensitivity of the chemo-resistant variant to IL-6/STAT3 signaling. Under basal conditions, we observed no difference in levels of pY705-STAT3 between Med8A-S and Med8A-R cells, however, treatment with the tyrosine phosphatase inhibitor, sodium vanadate, revealed a noticeable and significant increase of pY705-STAT3 in Med8A-R cells (Fig. 2e), suggesting the chemoresistant line have a low albeit intrinsically enhanced level of STAT3 activity.

### Loss of STAT3 or IL6R in drug resistant MB led to restored sensitivity to vincristine

**Fig. 3:**
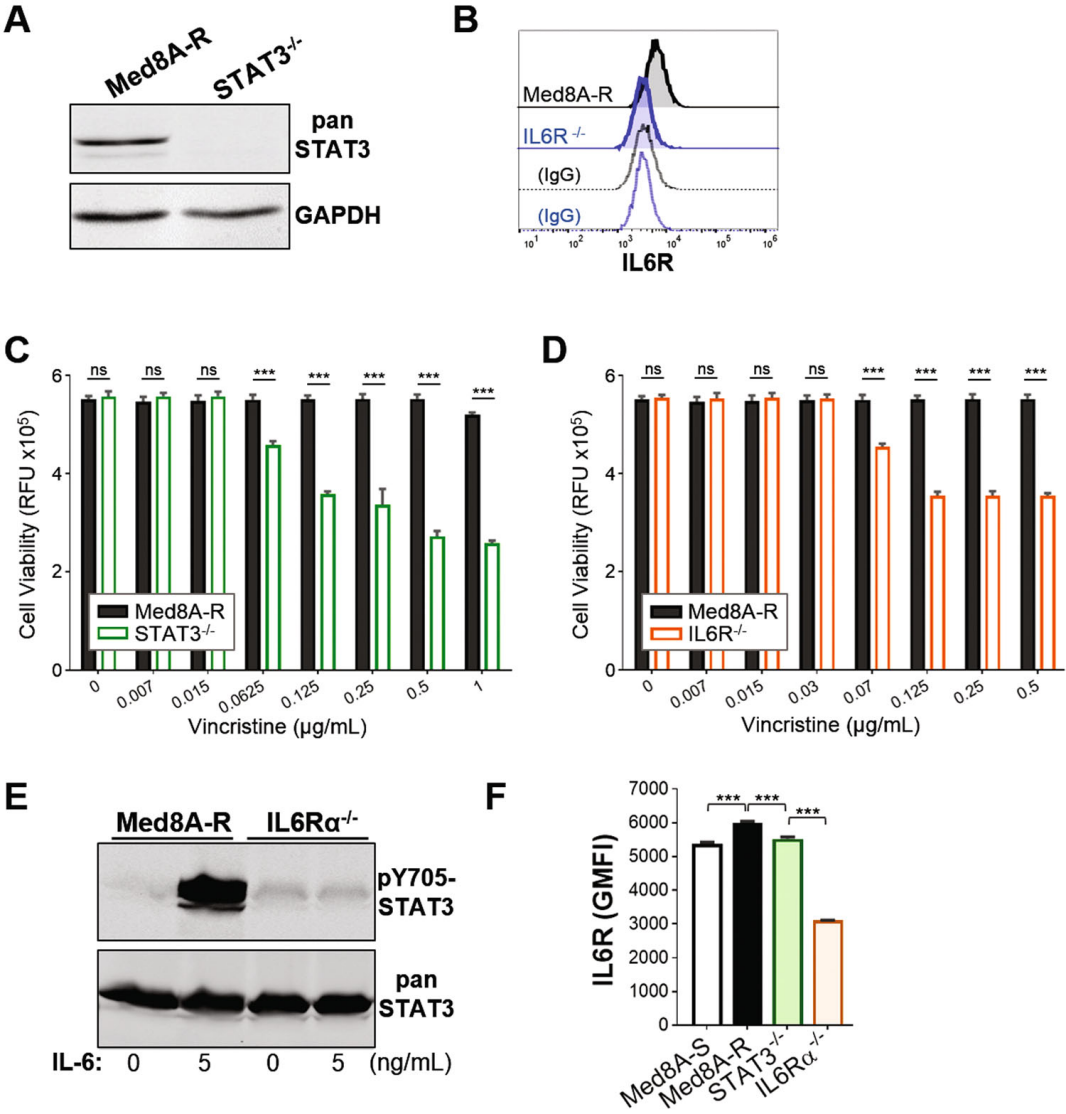
Loss of STAT3 or IL6R restores chemosensitivity in Med8A-R cells. **a** Western blot analysis of lysates of Med8A-R and STAT3^−/−^ cells for STAT3 and GAPDH expression. **b** Flow cytometry analysis of Med8A-R and IL6Rα^−/−^ cells for cell surface expression of IL6R, with corresponding IgG controls. Cell viability assay to assess the sensitivity of (**c**) STAT3^−/−^ and Med8A-R and (**d**) IL6R^−/−^ and Med8A-R cells to vincristine. As plotted is the mean ± SD of an experiment performed in triplicates, representative of at least 3 independent experiments. ***p < 0.001, two-way ANOVA with Bonferroni’s multiple comparison test. **e** Lysates of Med8A-R and IL6Rα^−/−^ untreated or treated with IL-6 were assessed for pY705-STAT3 and total STAT3 by western blot analysis. **f** Flow cytometry analysis of IL6R expression of Med8A-S, Med8A-R, STAT3^−/−^, and IL6R^−/−^ cells. As plotted is the mean ± SD of the geometric mean fluorescence intensity (GMFI) for an experiment performed in triplicates; ***p < 0.001, one-way ANOVA with Bonferroni’s multiple comparison test.

To further assess the requirement of STAT3 and IL-6 in drug resistance, we used CRISPR-Cas9 gene editing to generate clonal STAT3^−/−^ and IL6Rα^−/−^ derivatives in the stably resistant Med8A-R cell background (Supplementary Fig. 2). Med8A-R cells lacking STAT3 or IL6R (receptor for IL-6) expression (Fig. 3a, b) showed increased sensitivity to vincristine when compared to the parental Med8A-R cells (Fig. 3c, d). In addition, treatment of IL6Rα^−/−^ cells with IL-6 failed to stimulate any increase in pY705-STAT3 levels (Fig. 3e), indicating loss of IL6R led to complete blockade of IL-6 mediated STAT3 activity. We assessed and found that Med8A-R cells expressed higher cell surface levels of IL6R compared to Med8A-S, suggesting the increased STAT3 activity observed was due to increased IL-6 receptor function (Fig. 3f). Furthermore, IL6R expression was lower in STAT3^−/−^ cells when compared to the parental Med8A-R, but remain adequately expressed relative to Med8A-S cells (Fig. 3f), suggesting that IL-6 activity cannot overcome loss of STAT3 in promoting drug resistance. In summary, MB cells with blockade of IL-6 stimulation of STAT3 activity exhibited increased susceptibility to vincristine treatment. Taken together, our results provide evidence that increased IL-6/STAT3 signaling enhances vincristine resistance in MB, and implicates the IL-6/STAT3 signaling axis as a novel therapeutic pathway to circumvent drug resistance.

### Conditioning with exogenous IL-6 is sufficient to promote acquired drug resistance

**Fig. 4:**
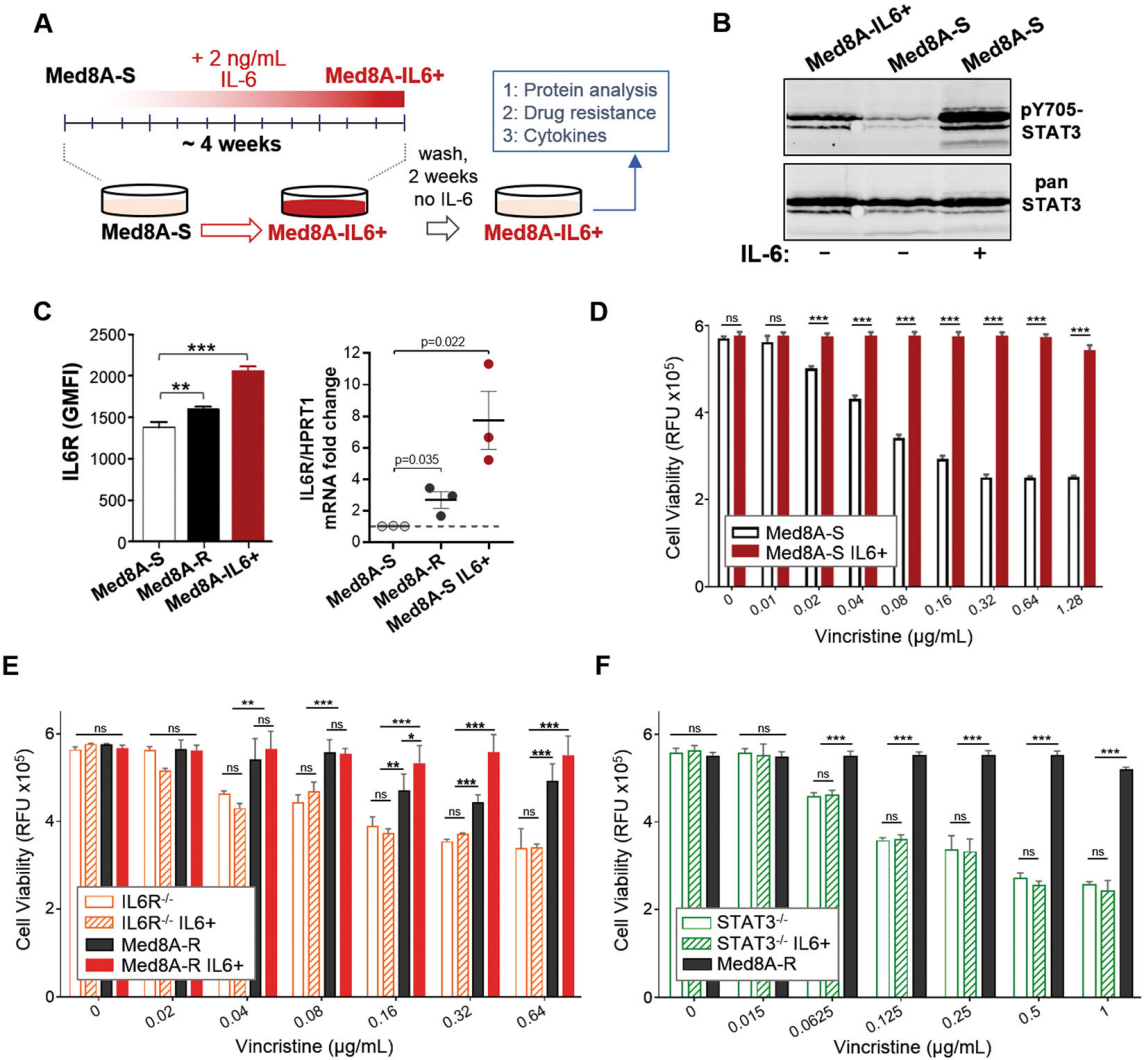
Exogenous IL-6 conditioning promotes drug resistance. **a** Schematic for IL-6 conditioning of MB cells. The chemosensitive Med8A-S cells were cultured for 4 weeks in media supplemented with 2 ng/mL IL-6. Following the conditioning stint, the cells were cultured a further 2 weeks without exogenous IL-6 to yield Med8A-IL6+ variant used for subsequent assays. **b** Western blot analysis of Med8A-S and Med8A-S-IL6+ cells for pY705 and total STAT3 levels under basal conditions, or transiently stimulated with 5 ng/mL IL-6 for 10 mins. **c** Analysis of Med8A-S, Med8A-R, and Med8A-S-IL6+ cells using flow cytometry for IL6R protein expression (Left panel), and QPCR for IL6R mRNA expression (Right panel). Error bars represent mean ± SD (*n* = 3); **p < 0.01, ***<0.001, two tailed unpaired t-test. Cell viability assay to assess the sensitivity of (**d**) Med8A-S and Med8A-S-IL6+ cells, (**e**) Med8A-R, Med8A-R-IL6+, IL6R^−/−^, and IL6R^−/−^-IL6+ cells, and, (**f**) Med8A-R, STAT3^−/−^ and STAT3^−/−^-IL6+ cells to vincristine. As plotted is the mean ± SD of an experiment performed in triplicates, representative of at least 3 independent experiments. ***p < 0.001, two-way ANOVA with Tukey’s multiple comparison test.

Given that incremental vincristine selection resulted in the resistant Med8A-R variant that exhibit enhanced IL-6/STAT3 activity, we postulated that constant exposure to IL-6 stimuli may be a sufficient driver of drug resistance in MB. To evaluate this, Med8A-S cells was conditioned with 2 ng/mL IL-6 for 4 weeks to derive the subline known as Med8A-IL6+ (Fig. 4a). Following the conditioning stint, Med8A-IL6+ cells were weaned off the exogenous IL-6 prior to further assays. At basal conditions, we found that Med8A-IL6+ cells exhibit increased pY705-STAT3 levels compared to the nonconditioned Med8A-S cells (Fig. 4b), with no detectable difference in total STAT3 expression (Supplementary Fig. 1). Med8A-IL6+ cells expressed correspondingly higher levels of IL6R protein and mRNA when compared to both Med8A-S and Med8A-R (Fig. 4c). Despite not having been selected with vincristine, we found that the relatively short term of conditioning with low levels of IL-6 was sufficient to render the Med8A-IL6+ cells highly resistant to vincristine (Fig. 4d), cisplatin, mitoxantrone, and idarubicin (Supplementary Fig. 3). We assessed and found that Med8A-S, Med8A-R, and Med8A-IL6+ cells proliferated at the same rates (Supplementary Fig. 4), thus the observed chemoresistance in Med8A-R and Med8A-IL6+ is not due to increased cell numbers alone. We do note that Med8A-R and Med8A-IL6+ exhibited an increased ability to grow clonally in vitro, suggestive of cooperative autocrine mediated effects (Supplementary Fig. 5). IL-6 conditioning of Med8A-R cells appeared to elevate vincristine resistance, but the difference was not always evident given the already resistant nature of these cells (Fig. 4e). IL-6 conditioning mediated drug resistance is dependent on IL6R as the receptor, since IL6Rα^−/−^ cells similarly conditioned with IL-6 exhibited no detectable enhancement of vincristine resistance (Fig. 4e). STAT3 is also required, since IL-6 conditioning of STAT3^−/−^ cells failed to enhance resistance to vincristine (Fig. 4f).

### Chemoresistant MB is susceptible to combination treatment of vincristine with cisplatin or niclosamide

**Fig. 5:**
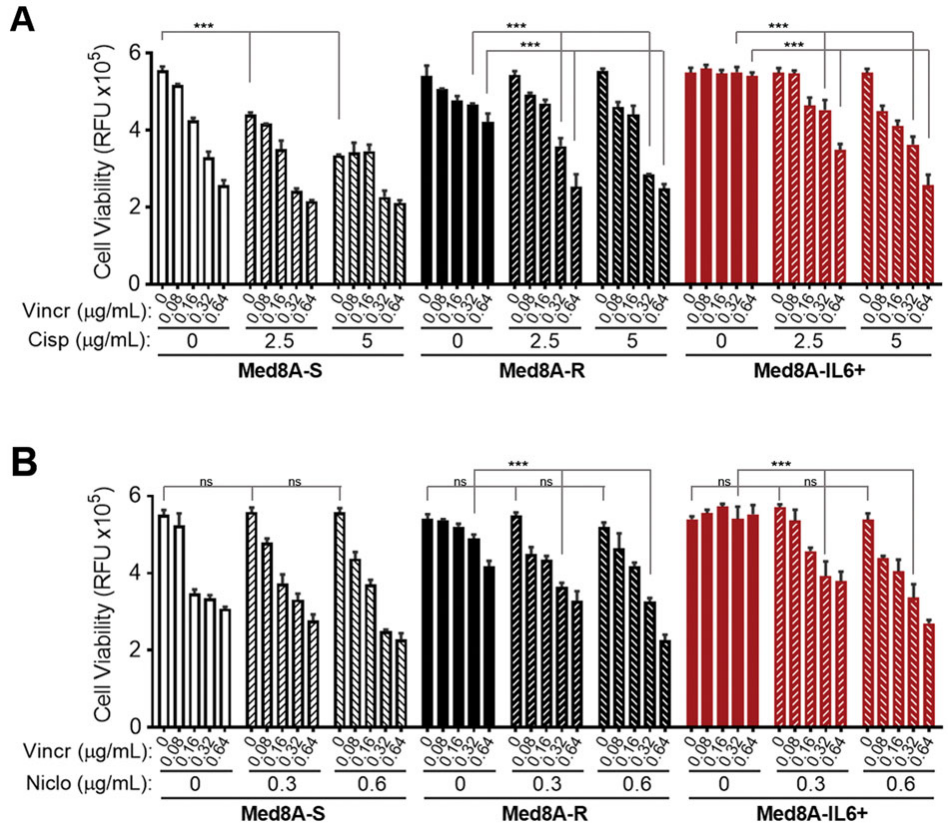
Chemoresistant MB is susceptible to combination treatment of vincristine with cisplatin or niclosamide. Med8A-S, Med8A-R, and Med8A-S-IL6+ cells were treated with vincristine alone or in combination with (**a**) cisplatin and (**b**) niclosamide at the indicated concentrations for 48 h and cell viability assessed with CTB. As plotted is the mean ± SD of three replicates; ***p < 0.001, two-way ANOVA with Tukey’s multiple comparison test (Significance not highlighted in the figure is presented in Supplementary Table 1).

Since Med8A-R and Med8A-IL6+ exhibited resistance to several drugs when given as a monoagent (Figs. 1, 4, Supplementary Fig. 3), we assessed if combined therapy may be able to overcome the observed resistance. As shown in Fig. 5a, combined treatment of vincristine and cisplatin effectively overcame the resistance observed for Med8A-R and Med8A-IL6+ to vincristine or cisplatin alone. In addition, we evaluated the effect of the selective STAT3 inhibitor, niclosamide, alone and in combination with vincristine. We assayed niclosamide at 0.3 and 0.6 µg/mL, since it was shown that concentrations below 1 µg/mL is subtoxic to healthy human neural stem cells^28^. As shown in Fig. 5b, Med8A-S, Med8A-R, and Med8A-IL6+ cells were not susceptible to low doses of niclosamide as a monoagent. However, when used in combination, niclosamide effectively overcame the resistance observed for Med8A-R and Med8A-IL6+ cells to vincristine (Fig. 5b). Our findings suggest that sub-toxic levels of a STAT3 inhibitor as well as another chemotherapeutic used in combination with lower concentrations of vincristine greatly enhances the susceptibility of chemoresistant MB.

### Autocrine IL-6 signaling promotes drug resistance in MB

**Fig. 6:**
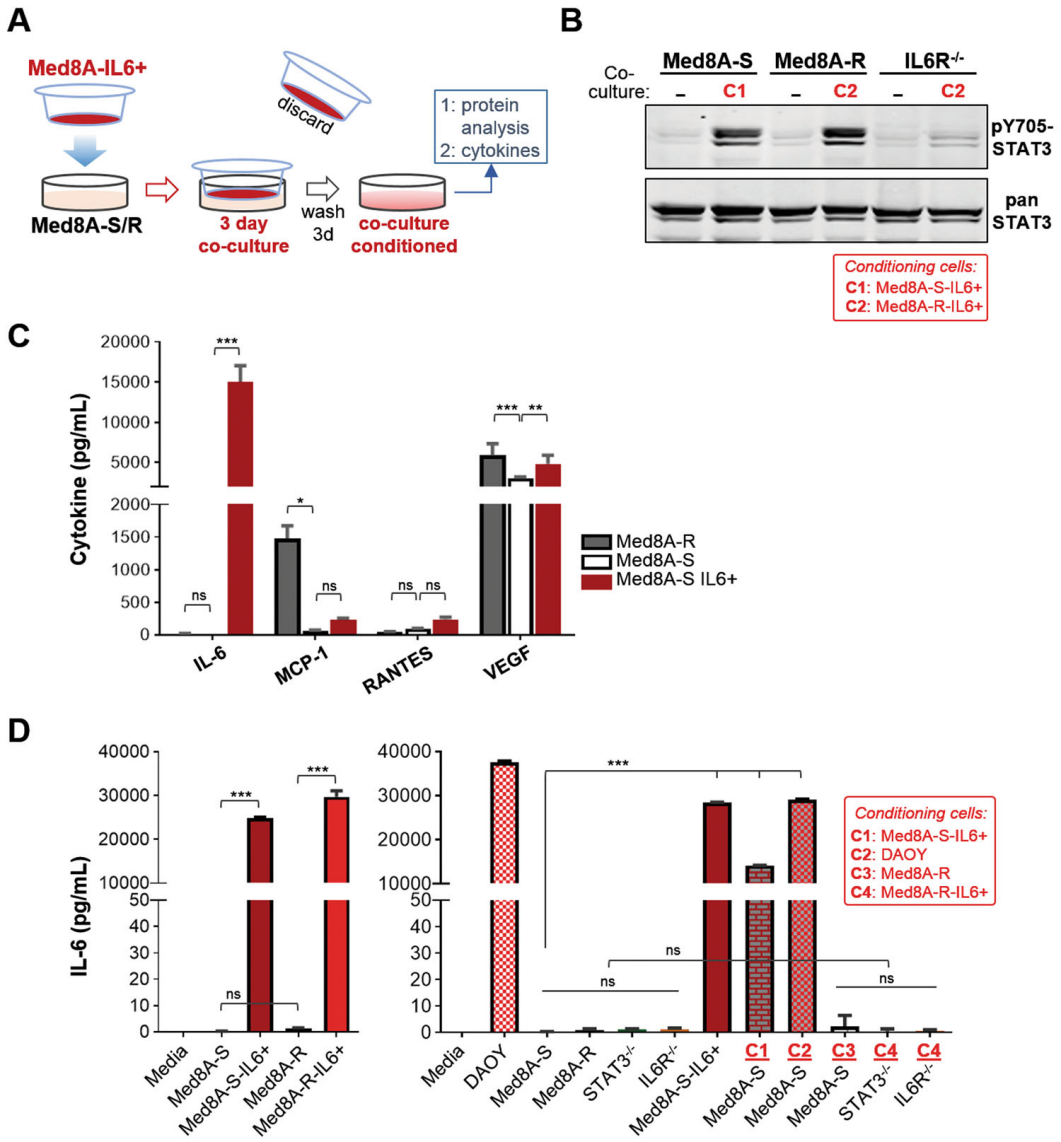
Autocrine IL-6 secretion promotes IL6/STAT3 activity. **a** Schematic of coculture system for cell-based conditioning of MB cells. Cells (e.g., Med8A-S) targeted for conditioning are plated in the bottom well, while pre-conditioned Med8A-IL6+ cells are plated in the top transwell insert with non-passable 1 µm pores that allow media exchange between the two cell populations. After 3 days in coculture, the insert is discarded, cells in the treatment well washed, and fresh media added (with no IL-6). After 3 days, cells are harvested for protein analysis while the conditioned media used for cytokine assays. **b** Western blot analysis for pY705 and total STAT3 levels of Med8A-S, Med8A-R and IL6R^−/−^ cells without (−) or with coculture treatment with Med8A-S-IL6+ (C1) or Med8A-R-IL6+ (C2) cells. **c** Secreted cytokine analysis of conditioned media from Med8A-S, Med8A-R, and Med8A-S-IL6+ cells. As shown are four cytokines, IL-6, MCP-1, RANTES, and VEGF, from the panel of 20 cytokines that achieved significant quantifiable levels. **d** Secreted IL-6 analysis of conditioned media using an ELISA-based kit for the various cells, as indicated. Coculture cells plated in the transwell inserts are as follows: Med8A-S-IL6+ (C1); DAOY (C2); Med8A-R (C3); Med8A-R-IL6+ (C4). As plotted is the mean ± SD of three replicates; ***p < 0.001, **<0.01, *<0.05, two-way ANOVA with Dunnett’s multiple comparison test.

To evaluate the possibility that IL-6 autocrine signaling in Med8A cells can promote drug resistance, we adopted a no-contact coculture system where a drug resistant derivative is used as cytokine “donor” cells to condition the target cell population. Nonconditioned Med8A-S or Med8A-R cells were plated in the bottom treatment chamber, while conditioned Med8A-S-IL6+ or Med8A-R-IL6+ cells were placed within hanging tissue culture inserts, which effectively separated the cells while enabling exchange of media and soluble factors within the system (Fig. 6a). In this manner, we found that Med8A-S and Med8A-R cells cocultured for 3 days with their corresponding IL-6 conditioned cells exhibited robust activation of pY705-STAT3 compared with the absence of coculture (Fig. 6b). As a control, IL6Rα^−/−^ cells remain nonresponsive to coculture conditioning by Med8A-IL6+ cells, suggesting that IL-6/IL6R is a potent cytokine stimulatory pathway promoting STAT3 activity (Fig. 6b).

Next, we assayed for cytokines as soluble autocrine factors released by the drug resistant cells. We profiled the culture supernatant of Med8A-S, Med8A-R, and Med8A-S-IL6+ cells using an antibody-based quantitative cytokine array platform. Of the 20 cytokines examined using the array, four achieved quantifiable levels that was deemed statistically significant (Fig. 6c). Not surprisingly, IL-6 levels were greatly elevated (over 10,000-fold) in the supernatant of Med8A-IL6+ cells when compared to Med8A-S or Med8A-R, indicating that exogenous IL-6 conditioning resulted in cells that secreted more IL-6. Compared to Med8A-S, Med8A-R cells secreted significantly higher amounts (over 20-fold) of MCP-1 (monocyte chemoattractant protein-1), but not of IL-6. Both Med8A-R and Med8A-S-IL6+ cells secreted significantly higher levels of VEGF (vascular endothelial growth factor) when compared to Med8A-S, but at no greater than twofold maximum difference. The levels of RANTES were not significantly different between the 3 cell types assayed.

The cytokine array analysis provided strong evidence that IL-6 is the most prominent cytokine released by IL-6 conditioned Med8A cells that can function in an autocrine manner. Thus, we focused analysis on secreted IL-6 found in the conditioned media. Under nonstimulated conditions, Med8A-S, Med8A-R, STAT3^−/−^, and IL6R^−/−^ cells secrete very low levels of IL-6, while DAOY secrete high levels of IL-6 (Fig. 6d). The IL-6 conditioned Med8A-S-IL6+ and Med8A-R-IL6+ cells appear to sustain increased IL-6 secretion even after removal of the IL-6 stimuli. Increased IL-6 secretion was also detected for Med8A-S cells that had been cocultured with either Med8A-S-IL6+ or DAOY cells, but not when cocultured with Med8A-R cells (Fig. 6d). Finally, neither STAT3^−/−^ nor IL6R^−/−^ cells respond to coculture mediated stimulation of IL-6 secretion, lending further support that an intact IL6R/STAT3 signaling pathway is required to sustain autocrine IL-6 activity. Taken together, our data provide strong evidence that IL-6 signals in an autocrine manner to promote increased pY705-STAT3 activity, IL-6 secretion and acquired drug resistance in Med8A cells.

### IL-6 conditioning leads to increased STAT3 activity, IL6R expression, and acquired drug resistance in other Group 3 MB cell lines

**Fig. 7:**
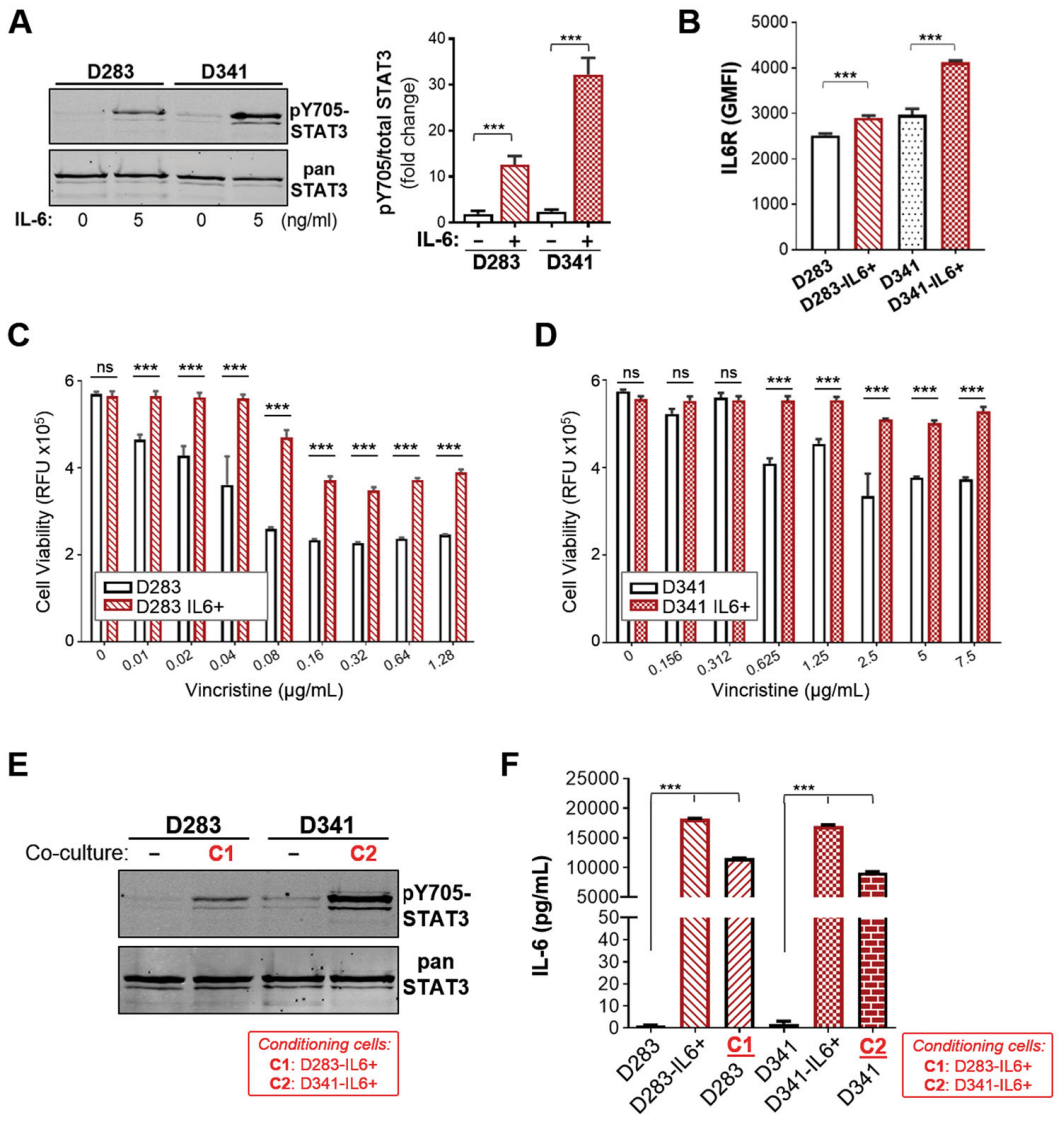
Group 3 MB cell lines D283 and D341 exhibit similar responses to IL6/STAT3 signaling. **a** D283 and D341 MB cells were treated with IL-6 at 5 ng/mL for 10 min and cell lysates immunoblotted for pY705 and total STAT3. Left panel: Representative blot of 3 independent replicates. Right panel: Quantitation of pY705-STAT3 over total STAT3 (mean ± SD, *n* = 3, ***p < 0.001, one-way ANOVA with Bonferroni’s post-test). **b** Flow cytometry analysis for expression of IL6R for D283 and D341 cells without and with IL-6 conditioning (as per Fig. 4a). As plotted is the GMFI mean ± SD of an experiment performed in triplicates, and representative of 3 independent experiments (***p < 0.001, one-way ANOVA with Bonferroni’s post-test). Cell viability assay to assess the sensitivity of (**c**) D283 and D283-IL6+ and, (**d**) D341 and D341-IL6+ cells to vincristine. As plotted is the mean ± SD of an experiment performed in triplicates, representative of 2 independent experiments (***p < 0.001), one-way ANOVA with Bonferroni’s multiple comparison test. **e** Western blot analysis for pY705 and total STAT3 levels of D283 and D341 cells without (−) or with coculture treatment with D283-IL6+ (C1) or D341-IL6+ (C2) cells, respectively. **f** Secreted IL-6 analysis of conditioned media from D283 or D341 cells, their IL-6 conditioned derivatives, and upon coculture with D283-IL6+ (C1) or D341-IL6+ (C2) cells. Plotted is mean ± SD of triplicate experiments; ***p < 0.001, two-way ANOVA with Dunnett’s multiple comparison test.

Our work with the Group 3 MB Med8A cell line has demonstrated a vital role of the IL-6/STAT3 pathway in the development of acquired drug resistance. To assess if this may be a generally applicable phenomenon, we profiled the expression of STAT3 and assessed the effects of IL-6 conditioning on D283 and D341, cell lines that also belong to Group 3 MB. Similar to Med8A cells, D283 and D341 cells exhibit low pY705-STAT3 levels at basal state, and that is rapidly inducible with bolus IL-6 treatment (Fig. 7a). Next, we subjected D283 and D341 to the same conditioning regime with low IL-6 to study the effects on acquired drug resistance. IL-6 conditioning of D283 and D341 cells resulted in significant increases in IL6R expression when compared to the nonconditioned cells (Fig. 7b). IL-6 conditioning of D283 and D341 cells also resulted in cells exhibiting enhanced resistance to vincristine treatment (Fig. 7c, d). Last, we assessed the ability of the drug resistant derivatives to condition the chemosensitive ones using the coculture model. When cocultured with their respective IL-6 conditioned cells, D283 and D341 exhibited increased pY705-STAT3 levels (Fig. 7e) and increased secretion of IL-6 (Fig. 7f). Hence, our data provide a consensus that Group 3 MB cell lines are highly responsive to IL-6 stimulation and promotion of STAT3 signaling that plays a prominent role in the development of acquired drug resistance in Group 3 cell lines.

### *STAT3* expression is significantly enriched in Group 3γ subtype MB

**Fig. 8:**
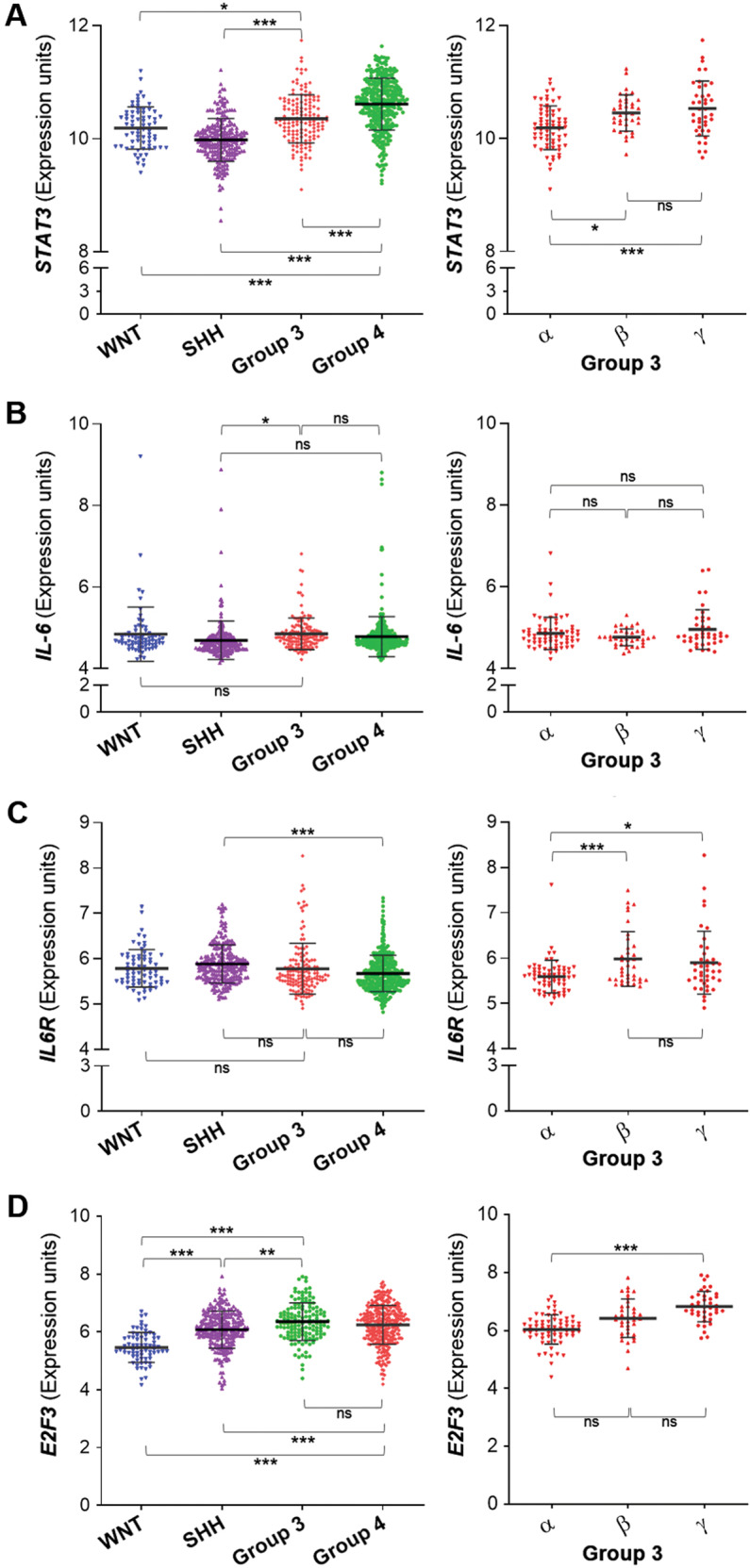
Expression profiling of STAT3, IL-6 and IL6R in subgroups of MB. The GSE85217 gene expression database comprises a cohort of 763 primary MB samples categorized into the 4 major subgroups WNT, SHH, Group 4 and Group 3. Group 3 is further subcategorized into 3α, 3β, and 3γ subtypes for the purposes of this analysis. Expression of (**a**) *STAT3*, (**b**) *IL-6*, (**c**) *IL6R* and (**d**) *E2F3* was analyzed according to their subgroup and subtype categorization. ***p < 0.001, **<0.01, *<0.05, one-way ANOVA with Tukey’s post-test.

Group 3 MB tumors are the most difficult to treat. To account for the high level of tumor heterogeneity, Cavalli et al. have classified Group 3 MB into additional subtypes, namely Groups 3α, 3β, and 3γ^29^. Most notable is Group 3γ that has high MYC amplification, high rates of metastasis, and worse overall survival. We analyzed the expression of select target genes relevant to this study using the GSE85217 gene expression database, comprising a cohort of 763 primary MB samples. *STAT3* was found to be enriched in Group 3/Group 4 when compared with SHH and WNT, subgroups that have a high overall survival and low metastatic incidence (Fig. 8a). Within Group 3, *STAT3* expression was higher for 3β and 3γ when compared to 3α. *IL-6* expression was significantly higher in Group 3 only when compared to SHH, but were otherwise not remarkable between the different major groups, nor between the Group 3 subtypes (Fig. 8b). *IL6R* expression was significantly higher in SHH when compared to Group 4, but were otherwise not different between all other subgroups (Fig. 8c). Within Group 3, *IL6R* expression was higher in 3β and 3γ when compared to 3α. In addition, we assessed the expression of *E2F3*, a transcription factor shown to directly bind the promoter of *IL6R* and regulate its expression^30^. *E2F3* expression is elevated in Group 3/Group 4 when compared to WNT and SHH. Within Group 3, *E2F3* is significantly higher in 3γ when compared to 3α (Fig. 8d). We also compared *E2F3* expression in our Med8A sublines, and found that both Med8A-R and Med8A-S-IL6+ cells have elevated *E2F3* mRNA expression when compared to Med8A-S (Supplementary Fig. 7). In summary, increased *STAT3* expression was correlated with increased *IL6R* in subtypes 3β and 3γ when compared to 3α, and suggestive of increased sensitivity to IL-6 cytokine stimulation of STAT3 activity.

## Discussion

Transcriptional profiling of MB has led to categorization of four distinct molecular subgroups. However, risk stratification and therapeutic intervention have yet to be refined to improve clinical outcome and long-term sequelae of MB patients. The conventional treatment approach is undermined by treatment refractory tumors and frequent occurrence of relapse, with high variability in survival outcome^1,2,4,6,8–10,31^. Specific targeted therapies are currently required to efficiently treat this aggressive malignancy^32^. Importantly, knowledge of activated signaling pathways in promoting treatment resistance and pathogenesis of the disease is key to designing novel therapeutic options.

Our study highlights a prominent role for IL-6 mediated activation of oncogenic STAT3 signaling able to give rise to acquired drug resistance in MB cell lines belonging to Group 3. In this context, IL-6 is a potential stimulatory cytokine found within the MB tumor microenvironment. We developed two model systems with a goal to identify a central pathway contributing to drug resistance. By subjecting the chemosensitive MB cell line, Med8A-S, to incremental selection with vincristine, we derived the variant Med8A-R that exhibited resistance to not only vincristine, but also to agents with different mechanisms of action. Characterization of this stably resistant derivative identified enhanced sensitivity to IL-6 mediated activation of STAT3, attributed in part to enhanced expression of IL6R. Loss of STAT3 or IL6R expression nullified the drug resistance of Med8A-R cells, indicating that IL-6/STAT3 signaling is a major driver of acquired drug resistance.

Notably, the chemoresistant DAOY cells exhibit constitutive pY705-STAT3 levels, while basal pY705-STAT3 in Med8A-R remains low, requiring IL-6 stimuli to provoke an enhanced response. This observation suggested that constant stimulation of the pathway may suffice to generate drug resistance. We confirmed this by using chronic low-level IL-6 stimuli to condition chemosensitive cells for 4 weeks, and found this method was highly effective in generating vincristine-resistant cells, despite not having been selected with the drug. Chemoresistance mediated by IL-6 conditioning similarly required IL6R or STAT3, again highlighting the indispensable nature of both proteins that likely function in linear fashion, with IL-6 as the extracellular upstream cytokine, IL6R as the receptor that activate JAKs, and subsequent phosphorylation and activation of STAT3.

Although the significance of STAT3 signaling in survival, proliferation and drug resistance is well known^17,33^, gaps remain in our understanding of the upstream regulators and activation of JAK-STAT3 signaling in MB. In particular, pY705-STAT3 has been linked to drug resistance in multiple malignancies, whereas pS727-STAT3 is required for maximal transcriptional activation^34^. Our study with Group 3 MB cell lines indicated that cells rendered drug resistant, either by drug selection or with sustained cytokine conditioning, exhibit low basal levels of pY705-STAT3 that remains inducible at enhanced levels by IL-6. Importantly, this mechanism invoked sustained activation of STAT3 dependent on elevated cytokine production, and not necessarily via mutational events of upstream regulators that result in constitutively activated STAT3. Our knockout studies highlight the requirement of not only STAT3 in drug resistance mediated by IL-6 conditioning, but that includes IL6R as a signaling intermediary.

STAT3 activity promotes upregulation of oncogenic downstream targets that include *cyclin D1*, c-*myc, bcl-2, survivin, bcl-XL*, and *VEGF*^35^. MYC amplification in a subset of Group 3 MB contributes to poor disease outcome^29^, and IL-6 is known to stimulate translation of c-Myc^36^. The MB cell lines used in this study exhibit high basal levels of c-Myc, and IL-6 stimulated significant increases of c-Myc in Med8A-S, Med8A-R and D341 (Supplementary Fig. 7). Interestingly, MYC amplified MB may be targeted with BET and HDAC inhibitors^37,38^, presenting a therapeutic option for chemoresistant MB driven by IL-6/STAT3 signaling.

Studies have shown that novel STAT3 inhibitors were able to disrupt pY705-STAT3 activity and suppress cell proliferation and growth in MB^22,23,39–41^. However, single pathway inhibition of STAT3 has led to feedback activation of other prominent survival pathways including EGFR and MEK/ERK, with ensuing reduced effectiveness of further STAT3 inhibition^42,43^. Similarly, IL-6 blockade therapy led to upregulation of EGFR signaling^44^. One strategy to mitigate development of resistance to any one agent is to use combination therapy. We showed that chemoresistant variants Med8A-R and Med8A-IL6+ cells were susceptible to combined vincristine and STAT3 inhibition with sub-toxic doses of niclosamide. Similarly, combined use of vincristine and cisplatin was effective at overcoming resistance observed for either agent when used as a monotherapy. Additional therapeutic options could include targeting of upstream or downstream components of the pathway, for example, inhibition of IL6R or JAK kinases in the IL-6/STAT3 pathway^42,43^, as well as coinhibition of other prominent pro-survival pathways such as EGFR/ERK.

Tumor cells have been known to exploit IL-6 to evade apoptosis by acquiring drug resistance through activation of pro-survival oncogenic pathways^45^. In our study, prolonged exposure to IL-6 promotes resistance to treatment in several Group 3 MB cell lines. IL-6 conditioned cells exhibit increased pY705-STAT3 activity, IL6R expression, elevated levels of IL-6 secreted and acquired resistance to vincristine. Our cytokine array assay also revealed increased levels of RANTES (CCL5), monocyte chemoattractant protein 1 (MCP1), and VEGF secreted by Med8A-IL6+ in response to IL-6 conditioning. RANTES is a chemokine that facilitates leukocyte infiltration and inflammation^46^. MCP1 promotes recruitment of immune cells in the tumor microenvironment^47^. VEGF is an angiogenic factor that facilitates the formation of blood vessels and supplies nutrients to the tumor cells. In sum, these cytokines are potential downstream targets of STAT3 known to promote tumorigenesis and metastasis in multiple malignancies^14,48–50^.

Our study also supports the notion that IL-6 can function in an autocrine fashion. IL-6 conditioned MB cells secrete high levels of IL-6, while increased expression of IL6R is indicative of an auto-feedback loop akin to oncogene-addiction, albeit one employing a cytokine-receptor pair. Similar mechanistic signaling has been reported to facilitate malignant transformation and activation of STAT3 in other tumors. Lung adenocarcinomas with activating mutations in EGFR was found to produce high IL-6 levels responsible for constitutive pY705-STAT3 activity^51^. In basal-like breast carcinomas, autocrine IL-6 sustains Notch-mediated promotion of proliferative self-renewal and increased invasiveness^52^.

Several clinically approved monoclonal antibodies and inhibitors have been developed to target IL6R and IL-6^53^. Due to the clinical correlation of increased levels of IL-6 in serum and poor prognosis of tumors, blocking the IL-6/STAT3 signaling axis could be beneficial in improving treatment refractory cancers^54–56^. Other Group 3 MB cell lines evaluated in our study exhibited a similar phenotype when exposed to IL-6 conditioning, including increased pY705-STAT3 activity, IL6R expression, IL-6 secretion and vincristine resistance.

Group 3 MB manifest as high-risk tumors due to MYC amplification and metastasis at diagnosis^7^. In light of our experimental findings, we analyzed the expression of *IL-6*, *IL6R*, *STAT3*, and *E2F3* in MB transcriptome databases to gain some clinical insights. Enriched *STAT3* levels is observed in Group 3 and Group 4 MB. Within the subgroup classification reported by *Cavalli* et al.^29^, Group 3γ subtype has the least favorable outcome, with high *MYC* amplification and frequent metastasis that correlated with increased *STAT3* expression. Higher *IL-6* expression in Group 3 over SHH also correlated with poor overall survival in Group 3 primary tumors. In contrast, *IL6R* levels was not significantly different between Group 3 and SHH. However, when considered within the subgroups, *IL6R* and *STAT3* levels in 3β and 3γ is significantly higher when compared to 3α, which correlated to Group 3 subtypes with worse outcomes. E2F3 is a transcription factor able to transactivate *IL6R* expression^30^ and we found that *E2F3* expression is elevated in Group 3γ MBs, and in our chemoresistant Med8A derivatives. In sum, our analyses revealed increased expression of key components involved in IL-6/STAT3 signaling in Group 3 MB.

In summary, our study demonstrated the functional consequence of targeting autocrine IL-6/STAT3 signaling in development of chemoresistance in Group 3 MB cell lines. We found that knocking out IL6R or STAT3 was sufficient to circumvent drug resistance, highlighting their potential for targeting in treatment of refractory MB. Our findings underscore how exogenous IL-6 was able to initiate an autocrine signaling machinery to evade drug-induced toxicity and promote sustained cell growth. MB cells exposed to and surviving chemotherapy, as well as cells conditioned to stimulatory cytokines present in the tumor microenvironment might pave the way to resistant clonal selection and constitutive activation of pro-survival pathways. Our study do not yet address the potential role of pro-inflammatory cells in the tumor microenvironment that may act via paracrine signaling to initiate and promote sustained cell growth and transformation in MB. Future studies could include investigation of brain tumor microenviromental cells that include tumor associated macrophages and microglia, T-lymphocytes, neutrophils, and astrocytes, as possible sources of IL-6 and other inflammatory cytokines^57^.

## Supplementary information

Supplementary Figure Legends

Supplementary Fig. 1

Supplementary Fig. 2

Supplementary Fig. 3

Supplementary Fig. 4

Supplementary Fig. 5

Supplementary Fig. 6

Supplementary Fig. 7

Supplemental Table Part1

Supplemental Table Part 2
