# Supplementary material for: Autocrine IL-6/STAT3 signaling aids development of acquired drug resistance in Group 3 medulloblastoma
Source: Cell Death Dis. 2020 Dec 5;11(12):1035. doi: 10.1038/s41419-020-03241-y (PMC7719195; doi:10.1038/s41419-020-03241-y)
Supplement: Supplementary file 1 — Supplementary Figure Legends [file 41419_2020_3241_MOESM1_ESM.docx]

**Supplementary Figure 1. Comparison of STAT3 expression in chemosensitive parental and chemoresistant derivatives of Med8A, D341 and D283 cell lines.**

Immunoblot of the indicated cell lysates were probed with antibodies against total STAT3 and GAPDH.

**Supplementary Figure 2. CRISPR-Cas9 generation of STAT3^-/-^ or IL6Rα^-/-^ in Med8A-R cells.**

(**A**) Immunofluorescence staining of Med8A-R (WT) and a clonal STAT3^-/-^ derivative for STAT3 (green) and nucleus (DAPI, blue). (**B**) Sequencing of the Med8A-R and STAT3^-/-^ cells revealed a homozygous 8bp missense deletion within the second coding exon of *STAT3* at the gRNA targeted site (gRNA underlined, PAM motif highlighted). (**C**) The 1^st^ coding exon of *IL6Rα* was similarly targeted using CRISPR-Cas9 in Med8A-R cells. As shown is the sequencing alignment for a single IL6Rα^-/-^ clone showing the indicated deletions within each allele.

**Supplementary Figure 3. IL-6 conditioning promotes resistance to cisplatin, mitoxanthrone and idarubicin.** Cell viability assay to assess the sensitivity of Med8A-S and Med8A-S-IL6+ cells to (A) cisplatin, (B) mitoxanthrone, and (C) idarubicin. As plotted is the mean +/- SD of an experiment performed in triplicates. P ***<0.001, **<0.01, two-way ANOVA with Bonferroni’s multiple comparison test.

**Supplementary Figure 4. Proliferation rate comparison of Med8A-S, Med8A-R and Med8A-IL6+ cells.** Cells were plated at initial seeding of 5000 cells per well in triplicates and proliferation monitored over 5 days using a live-imaging platform. As plotted are the phase area confluence (Mean +/- SD, n=3). The doubling time (in days) was derived from plotting an exponential growth curve; Med8A-S (1.111), Med8A-R (1.097) and Med8A-IL6+ (1.131).

**Supplementary Figure 5. Colony formation assay for Med8A-S, Med8A-R and Med8A-IL6+ cells.** Cells were plated at 200 cells per well and colony formation monitored over 7 days using a live-imaging platform. As plotted are the colony counts per well (minimum 25 cells per colony) at 7 days for triplicate experiments (Mean +/- SD, P ***< 0.001, **<0.01, two-tailed unpaired t-test).

**Supplementary Figure 6.** QPCR analysis of Med8A-S, Med8A-R and Med8A-S-IL6+ for *E2F3* mRNA expression. As plotted is the mean +/- SD of an experiment performed in triplicates. Two-tailed unpaired t-test.

**Supplementary Figure 7. IL-6 stimulation of c-Myc expression.** (**A**) The indicated cells were untreated or treated with 10 ng/mL IL-6 for 15 mins and lysates immunoblotted for pY705-STAT3, total STAT3, c-Myc and GAPDH. As shown is representative of 3 independent replicates. (**B**) Quantitation of c-Myc over GAPDH, reflected as fold change, from the data shown in **A** (Mean +/- SD, one-way ANOVA with Bonferroni’s post-test).
