# Supplementary figures and images for: Autocrine IL-6/STAT3 signaling aids development of acquired drug resistance in Group 3 medulloblastoma
Source: Cell Death Dis. 2020 Dec 5;11(12):1035. doi: 10.1038/s41419-020-03241-y (PMC7719195; doi:10.1038/s41419-020-03241-y)

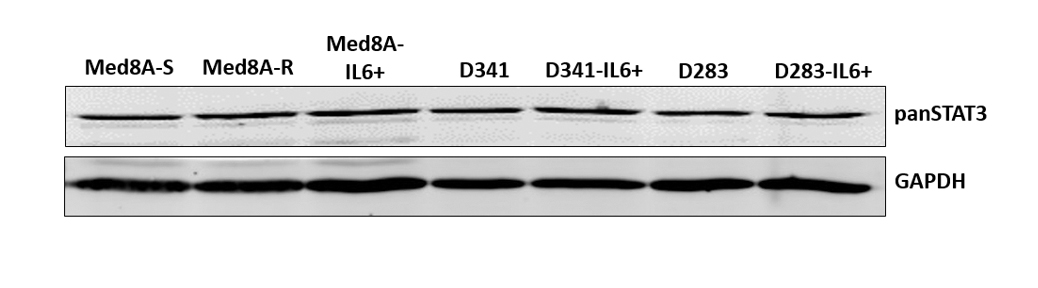

Supplement: Supplementary file 2 — Supplementary Fig. 1 [file 41419_2020_3241_MOESM2_ESM.jpg]

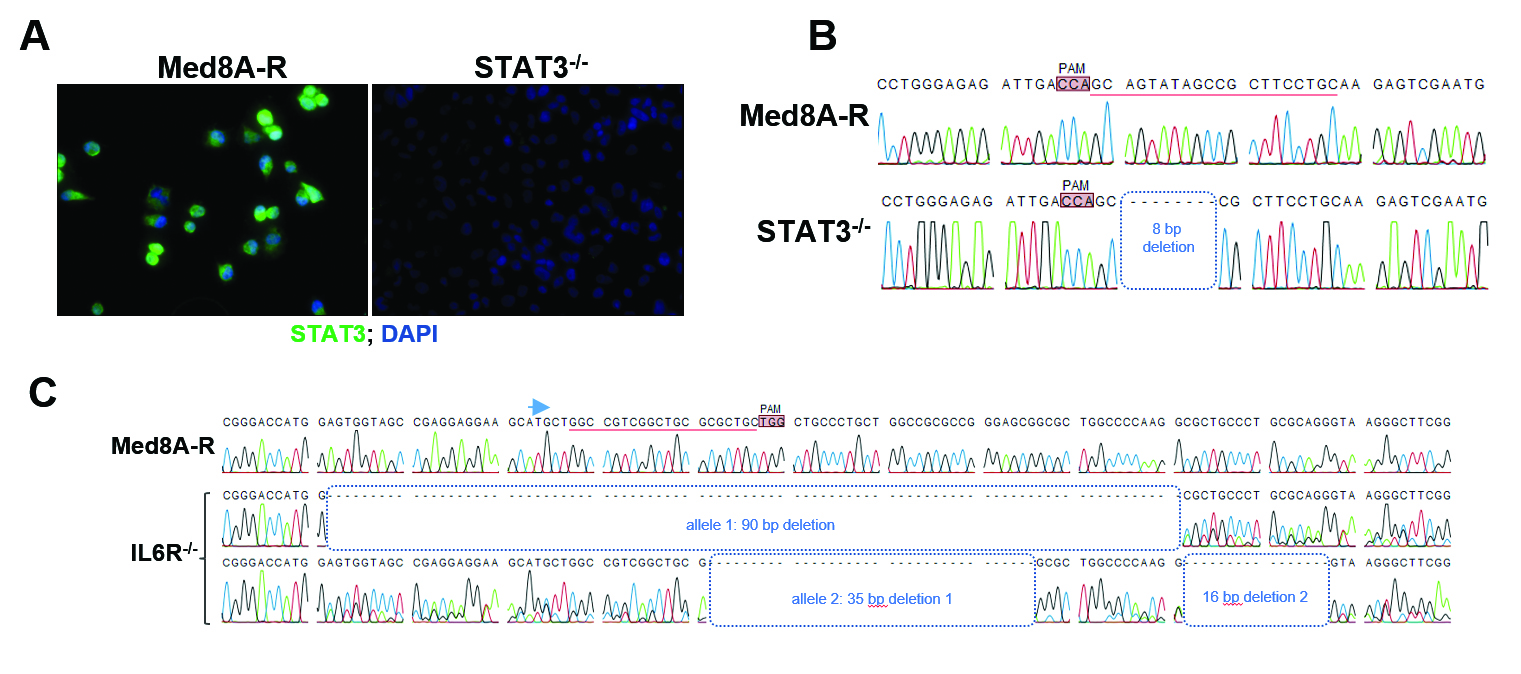

Supplement: Supplementary file 3 — Supplementary Fig. 2 [file 41419_2020_3241_MOESM3_ESM.jpg]

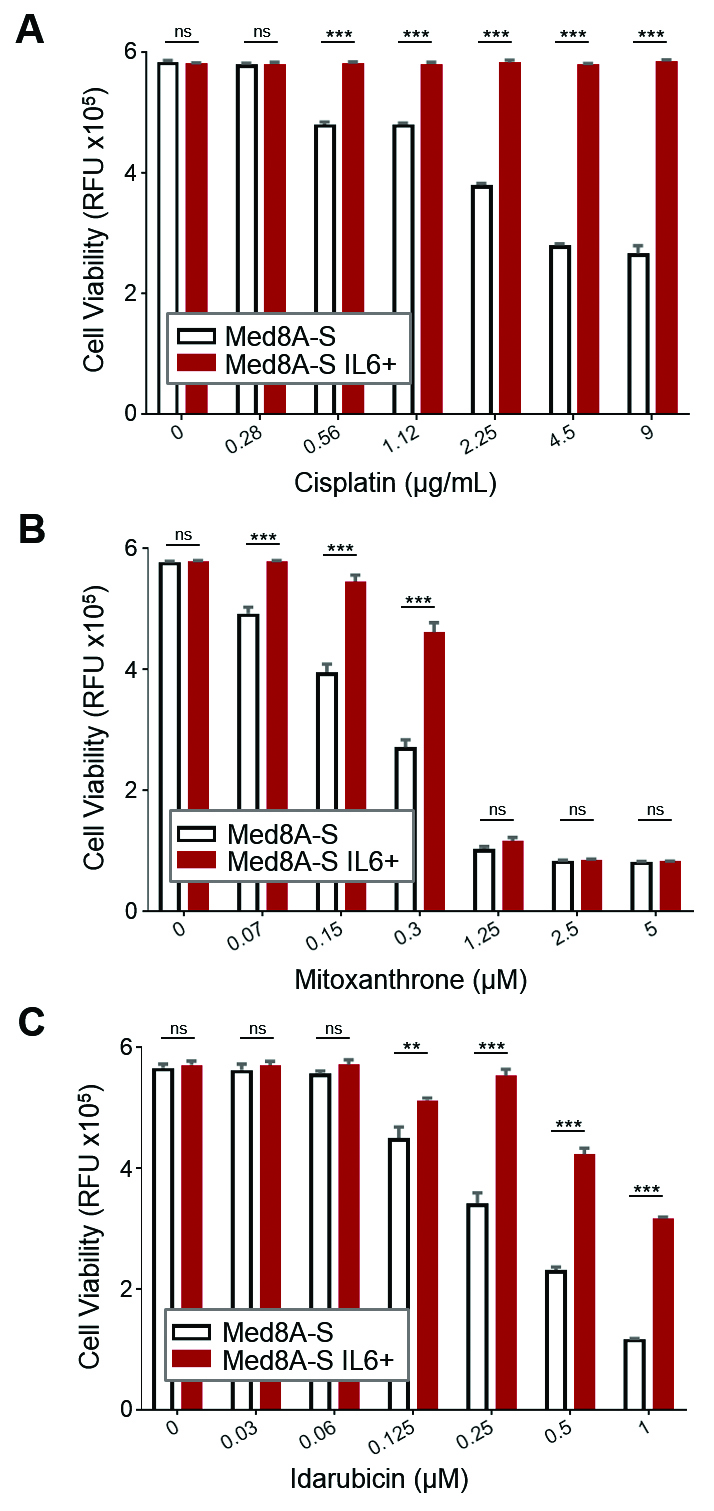

Supplement: Supplementary file 4 — Supplementary Fig. 3 [file 41419_2020_3241_MOESM4_ESM.jpg]

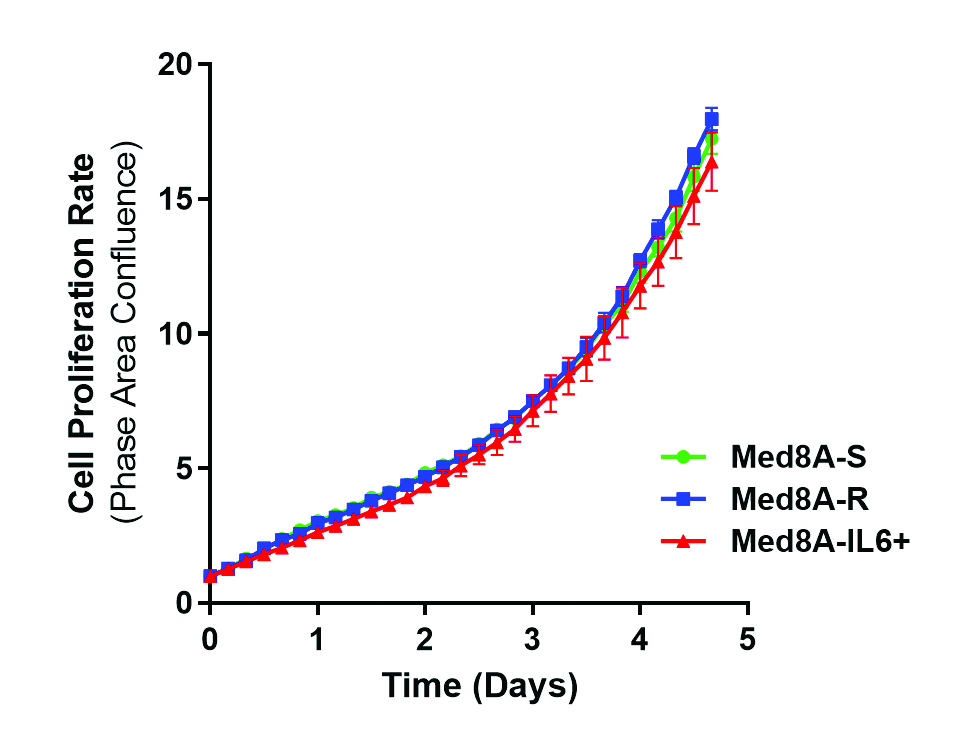

Supplement: Supplementary file 5 — Supplementary Fig. 4 [file 41419_2020_3241_MOESM5_ESM.jpg]

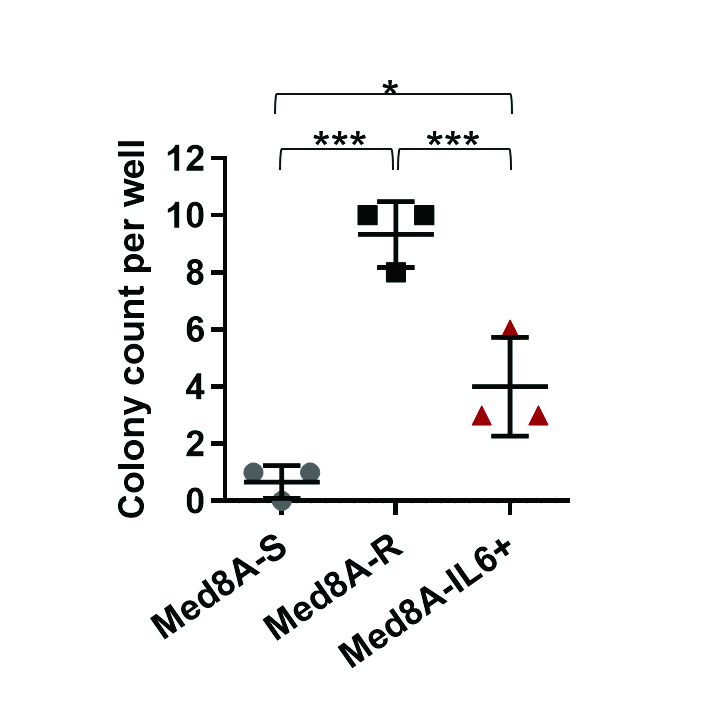

Supplement: Supplementary file 6 — Supplementary Fig. 5 [file 41419_2020_3241_MOESM6_ESM.jpg]

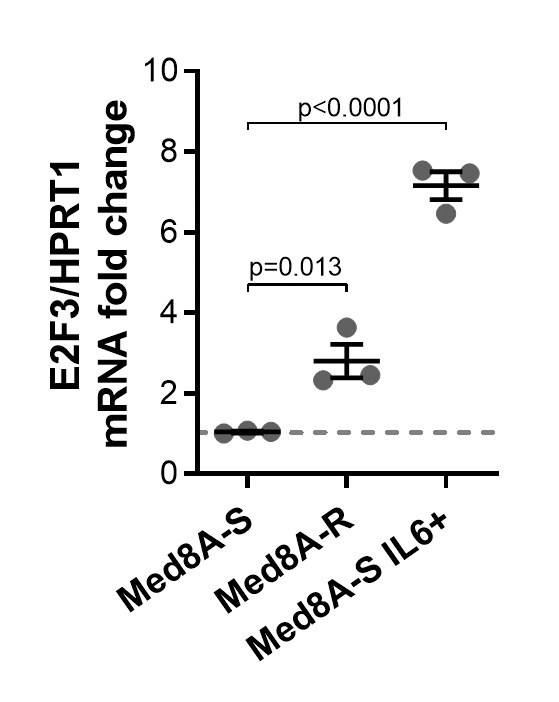

Supplement: Supplementary file 7 — Supplementary Fig. 6 [file 41419_2020_3241_MOESM7_ESM.jpg]

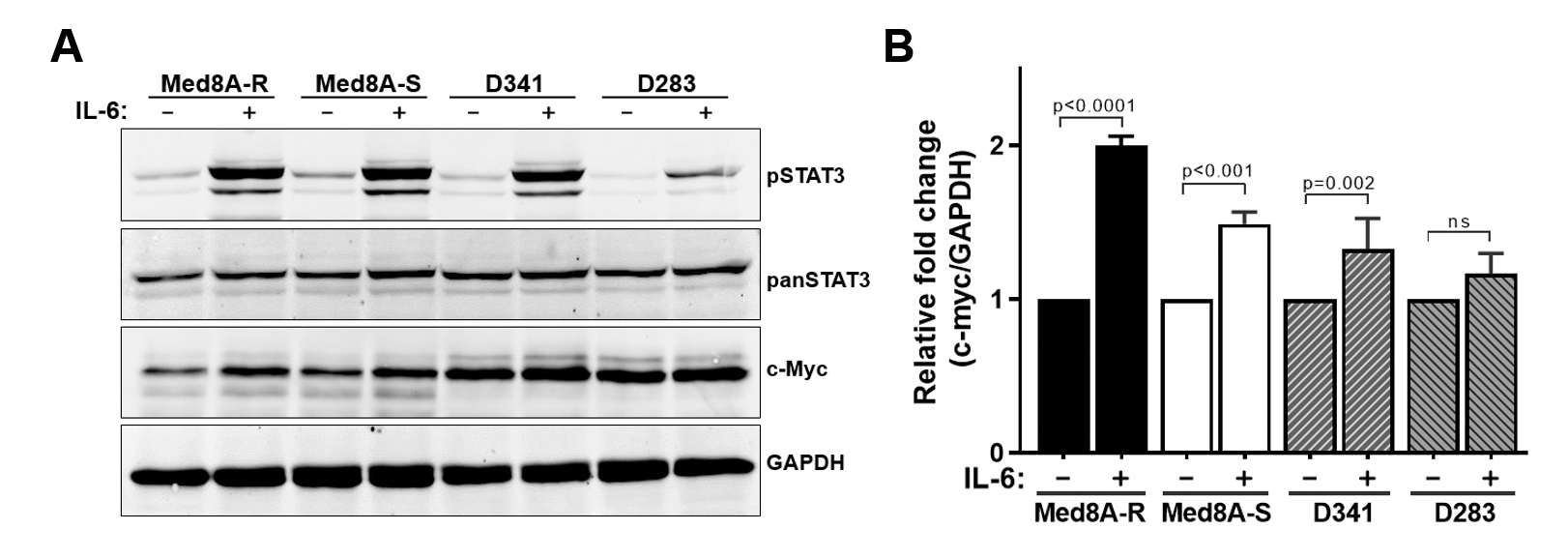

Supplement: Supplementary file 8 — Supplementary Fig. 7 [file 41419_2020_3241_MOESM8_ESM.jpg]
